# Cost and cost effectiveness of geospatial planning and delivery tools added to standard health campaigns in Luapula Province, Zambia

**DOI:** 10.1093/oodh/oqae040

**Published:** 2024-12-02

**Authors:** Anna Winters, Christina Riley, Livashan Soobramoney, Derek Pollard, Elizabeth Jere, Frazer Bwalya, Kafula Silumbe, Busiku Hamainza

**Affiliations:** Akros Inc., Timberlane, Missoula, MT 59802, USA; Akros Research, 45A Roan Road, Lusaka, Zambia; Akros Research, 45A Roan Road, Lusaka, Zambia; Akros Research, 45A Roan Road, Lusaka, Zambia; Akros Research, 45A Roan Road, Lusaka, Zambia; Akros Research, 45A Roan Road, Lusaka, Zambia; PATH, Chainama Hills Hospital Grounds, Lusaka, Zambia; National Malaria Elimination Centre, Republic of Zambia Chainama Hills Hospital, Lusaka, Zambia

**Keywords:** geospatial, impact, return on investment, malaria, digital, precision health, indoor residual spray

## Abstract

Geospatial tools are used to map populations to support microplanning and delivery of health campaigns. Although the value of geospatial tooling has been described, their costs and cost effectiveness is largely unknown. This study details the results of a cost-effectiveness analysis of a digital geospatial tool (‘Reveal’) added to a 2017 malaria control campaign [indoor residual spraying (IRS)] in Zambia. An economic evaluation of the costs for digital geospatial tooling to microplan and deliver IRS for malaria in Luapula Province, Zambia, was conducted using primary data collection methods in line with a recently developed methodology termed ‘Total Cost of Ownership’. A cost-effectiveness estimate was calculated for adding the geospatial tooling to standard IRS scaling over 5 years. Results indicate that use of Reveal attributed an average 21% reduction in cost per case averted (Ca) compared to IRS alone. Cost per Ca with IRS alone was estimated at $18.16 compared to cost per Ca when the geospatial tooling was added ($15.51 in year 1, $13.93 by year 5). Savings per Ca were realized through use of Reveal during IRS campaign deployment, likely through the mechanism of the tool, which supports field teams to use digital maps to find and spray houses. Analysis of current and ongoing cost for deployment does warrant further consideration and investment toward digitized geospatial tooling, especially considering the bearing these tools have on multiple health campaigns, globally. Further consideration on scaling strategies and expansion to other health campaigns and applications is also warranted.

**RESUMEN:**

Las herramientas geoespaciales se usan para ubicar poblaciones y apoyar la microplanificación y la distribución de campañas de salud. Aunque se ha descrito el valor de las herramientas geoespaciales, sus costos y la efectividad de los costos se desconocen en gran medida. Este estudio desglosa los resultados de un análisis de efectividad de costos de una herramienta geoespacial (‘Reveal’) agregada a una campaña de control de la malaria de 2017 (fumigación residual en interiores, [IRS por sus siglas en inglés]) en Zambia. Se llevó a cabo una evaluación de los costos para herramientas geoespaciales digitales con el propósito de microplanificar y distribuir la IRS para malaria en la provincia de Luapula, Zambia, usando métodos de recolección de datos primarios, alineados con una metodología desarrollada recientemente conocida como ‘Costo total de propiedad’. Se calculó un estimado de efectividad de costos respecto a agregar la herramienta geoespacial a IRS estándar con escalamiento a lo largo de 5 años. Los resultados muestran que el uso de Reveal contribuyó a una reducción promedio de 21% en el costo por caso evitado (CA, por sus siglas en inglés) en comparación con la IRS por sí sola. El costo por Ca con IRS por sí sola se estimó en $18.16, en comparación con el costo por Ca cuando se agregó la herramienta geoespacial ($15.51 el primer año, $13.93 para el quinto año). Los ahorros por Ca se cumplieron mediante el uso de Reveal durante la implementación de la campaña de IRS, posiblemente a través del mecanismo de la herramienta, el cual apoya a los equipos en campo que usan mapas digitales para encontrar y fumigar casas. El análisis de los costos actuales y en curso justifica una mayor consideración e inversión en herramientas geoespaciales digitalizadas, considerando especialmente el efecto de estas herramientas en múltiples campañas de salud a nivel global. También se justifica una mayor consideración en estrategias y expansión del escalamiento hacia otras campañas de salud y aplicaciones.

**RESUMO:**

As ferramentas geoespaciais são utilizadas para cartografar as populações, e apoiar o microplaneamento e a realização de campanhas de saúde. Embora o valor das ferramentas geoespaciais tenha sido descrito, os seus custos e a sua relação custo-eficácia são amplamente desconhecidos. Este estudo detalha os resultados de uma análise de custo-eficácia de uma ferramenta geoespacial digital (‘Reveal’) adicionada a uma campanha de controlo da malária de 2017 (pulverização residual interna, PRI) na Zâmbia. Foi realizada uma avaliação económica dos custos das ferramentas geoespaciais digitais para microplaneamento e aplicação da pulverização residual intradomiciliária contra a malária na província de Luapula, na Zâmbia, utilizando métodos de recolha de dados primários em conformidade com uma metodologia recentemente desenvolvida denominada ‘Custo Total de Propriedade’. Foi calculada uma estimativa da relação custo-eficácia para a adição das ferramentas geoespaciais ao escalonamento padrão da PRI durante cinco anos. Os resultados indicam que a utilização do Reveal atribuiu uma redução média de 21% no custo por caso evitado (CE) em comparação com o PRI isolado. O custo por CE apenas com o PRI foi estimado em 18,16 dólares em comparação com o custo por CE quando as ferramentas geoespaciais foram adicionadas (15,51 dólares no primeiro ano, 13,93 dólares no quinto ano). Foram realizadas poupanças por CE através da utilização do Reveal durante a implementação da campanha de PRI, provavelmente através do mecanismo da ferramenta que apoia as equipas de campo na utilização de mapas digitais para encontrar e pulverizar casas. A análise do custo atual e contínuo da implantação justifica uma maior consideração e investimento em ferramentas geoespaciais digitalizadas, especialmente tendo em conta a influência que estas ferramentas têm em múltiplas campanhas de saúde, a nível mundial. Também se justifica uma análise mais aprofundada das estratégias de expansão e alargamento a outras campanhas e aplicações no domínio da saúde.

**RÉSUMÉ:**

Les outils géospatiaux sont utilisés pour cartographier les populations afin de soutenir la microplanification et la mise en œuvre des campagnes de santé. Bien que la valeur des outils géospatiaux ait été décrite, leurs coûts et leur rentabilité sont largement inconnus. Cette étude détaille les résultats d’une analyse coût-efficacité d’un outil géospatial numérique (“Reveal”) ajouté à une campagne de lutte contre le paludisme de 2017 (pulvérisation intradomiciliaire d’insecticides, PID) en Zambie. Une évaluation économique des coûts des outils géospatiaux numériques pour la microplanification et la mise en œuvre de la PID contre le paludisme dans la province de Luapula, en Zambie, a été réalisée à l’aide de méthodes de collecte de données primaires conformément à une méthodologie récemment développée appelée “Coût total de possession”. Une estimation du rapport coût-efficacité a été calculée pour l’ajout de l’outil géospatial à la mise à l’échelle standard de la PID sur cinq ans. Les résultats indiquent que l’utilisation de Reveal a permis une réduction moyenne de 21% du coût par cas évité (CE) par rapport à la PID seule. Le coût par CE avec la PID seule a été estimé à 18,16 $ par rapport au coût par CE lorsque l’outil géospatial a été ajouté (15,51 $ la première année, 13,93 $ la cinquième année). Des économies par CE ont été réalisées grâce à l’utilisation de Reveal lors du déploiement de la campagne de PID, probablement grâce au mécanisme de l’outil qui aide les équipes de terrain à utiliser des cartes numériques pour trouver et pulvériser les maisons. L’analyse des coûts actuels et continus du déploiement justifie une réflexion plus approfondie et des investissements dans les outils géospatiaux numérisés, en particulier compte tenu de l’influence de ces outils sur de multiples campagnes de santé à l’échelle mondiale. Une réflexion plus approfondie sur les stratégies de mise à l’échelle et l’extension à d’autres campagnes et d’autres applications de santé est également justifiée.

## INTRODUCTION

**Figure 1:**
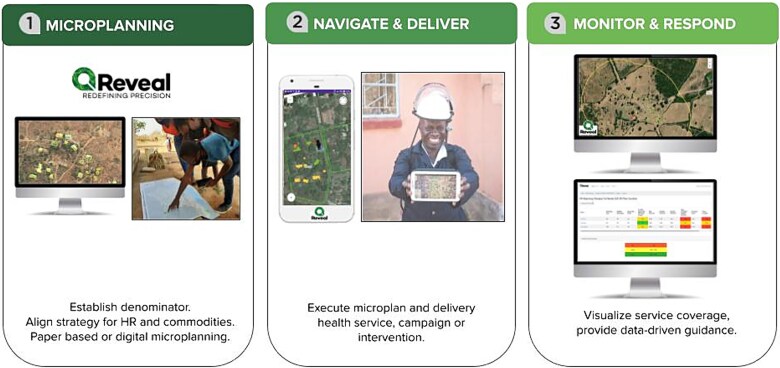
Three processes are supported by Reveal: digital maps and microplans are developed in step 1; plans are then tasked to field teams in step 2 to guide them to specific target areas and households and record intervention data; and in step 3, coverage and campaign progress data are visualized on dashboards, and used for campaign monitoring and mop-up

Malaria control and prevention efforts funded by national programs, global, multilateral and bilateral partners have focused on two primary tools, indoor residual spray (IRS) and long-lasting insecticide treated nets (LLINs). The World Health Organization (WHO) recommends that IRS or LLINs are utilized in high-malarious areas, sometimes both measures being applied [1, 2]. IRS is the application of insecticide to indoor walls of houses within malaria endemic areas to kill malaria vectors and reduce risk of malaria transmission [2]. Prior to an IRS campaign, central and local management teams conduct microplanning to identify the target areas, and plan for the staffing and commodities required to deploy IRS. Following microplanning, field teams visit houses to spray the interior walls of each sleeping structure. Both IRS and LLIN deployment represent a significant investment. The President’s Malaria Initiative (PMI) partners with 27 countries and has provided over 6.3 billion United States dollars (USD) since 2005 toward malaria control. Similarly, global fund has invested over 17 billion USD in malaria control programs since 2002 [3]. These costly malaria interventions require precise pre-planning and programmatic measurement to ensure appropriate population coverage is achieved and money is well-spent. WHO suggests a successful IRS campaign should have operational IRS coverage of at least 80% of eligible structures in areas targeted for IRS [2]. However, several studies that review IRS coverage showed that field teams conducting IRS campaigns frequently underestimate total households within targeted areas due to poor population data and substandard monitoring practices [4]. Paper forms have been a typical approach to data collection for IRS campaigns where spray teams collect data at each household visited, and these paper data are then collated during the campaign to estimate spray coverage and completion rates [2]. Martin et al. [5] detailed how a digital geospatial tool, termed Reveal (Fig. 1), supports national and local teams to microplan their IRS (as well as other campaign) operations, and monitors daily operations in the field. The Reveal platform captures and utilizes data on individual household structures and their geolocations to support IRS (and other health campaigns where applicable) through mapping and microplanning to the household or community level to plan campaign locations, logistics and required resources. Once the microplan is complete, the mobile phone application supports field teams to navigate and collect data on households as they deliver health services. Data are displayed on real-time dashboards describing the campaign or routine health service coverage.

Reveal was originally developed alongside the Zambia National Malaria Control Program’s (NMCP’s) efforts to deploy malaria interventions—particularly IRS [6]—and has since been used to support multiple campaign and intervention types including vaccination, behavioral change communication, seasonal malaria chemoprophylaxis, focus investigation, larval source management, and mass drug administration for malaria and also neglected tropical diseases.

As Reveal use has expanded, there has been a lack of costing data to understand its return on investment (ROI). There is limited costing data for other types of digital tooling as well, likely due to lack of funding, lack of time or lack of programmatic focus. Generally, as global health leaders seek to push digitalization forward, this paucity of data demonstrating the value of digital data capture in public health must be rectified [7]. ROI data for digital systems is necessary for donors and countries in the global south given the challenging situation of determining how to best spend often limited resources [8]. Further, there has been limited data available to support the value proposition for geospatial tooling (like that of Reveal) that uses satellite imagery to map populations and visualize health data to provide insight into the distribution of disease and responsive actions [9–11]. The Zambia Ministry of Health (MOH) and its partners are interested in high ROI strategies to maximize their investments. In this analysis, the costs and cost effectiveness in relation to disease cases averted (Ca) is described and reported through sharing the results of the digital geospatial tool, Reveal, added to the standard malaria control campaign (IRS) in Luapula Province, Zambia.

## Study area: Luapula Province, Zambia

Malaria is the main driver of morbidity and mortality in Zambia, particularly among children under the age of five [12]. Malaria has adverse socioeconomic impact and significantly exacerbates poverty. Given the availability of costing and campaign coverage data, coupled with the ongoing need for IRS delivery, the focus area for this assessment was Luapula Province, in northern Zambia. Luapula is located in the marshlands of the Luapula River bordering Lake Mweru [13]. The 2022 census recorded a population of 1.5 million in Luapula Province. This province consistently reports some of the highest malaria prevalence (600 to 700/1000 per year) despite routine malaria intervention campaigns [14]. Fishing and agriculture are the primary means of livelihood in this area. The main malaria vectors in Luapula Province (*Anopheles funestus* s.s. and *Anopheles gambiae* s.s.) exhibit a preference for feeding on humans and typically bite and rest indoors, hence the focused use of IRS and LLIN strategies [15, 16].

## MATERIALS AND METHODS

### Digital data collection implementation and setting

The goal of the analysis was to compare the cost and cost per Ca for the [IRS only] strategy to the [IRS + Reveal] strategy during the 2017 IRS campaign in Luapula Province, Zambia. During this campaign, Reveal was used by district and national managers to support the microplanning and delivery of the insecticide (Actellic 300CS) by local field teams. This strategy was designated [IRS + Reveal]. Based on estimated costs to implement Reveal in one district, costs were then estimated to scale Reveal to all of Luapula Province (11 additional districts) through expanding into 25 new health facility catchments (HFCs) each year across a period of 5 years until the entire province (and all rural health centers) were covered. These costs were compared to the [IRS only] strategy which was standard IRS deployment covering the same population. Analysis steps are summarized and listed here, and the data sources and design are described below.

### Cost and cases averted by [IRS only]

The Malaria INtervention Tool (MINT) was applied to estimate the number of malaria Ca through use of malaria interventions (IRS) in the target area without use of the geospatial digital tool, as well as the associated costs for IRS and cost per Ca. Parameters entered into the MINT model included a 5-year time period, scaling by 25 HFCs each year and up to a total of 125 HFCs total. MINT was developed by Imperial College to support NMCPs to explore cost-effective current WHO recommended insecticide treated nets (ITNs) and/or IRS products malaria control. Monitoring and evaluation (M&E) costs were not factored into the model (personal communication, T. Churcher and E. Sherrard-Smith), hence an additional 6.2% was added to the cost per Ca to factor in M&E [17, 18]. This figure was derived from a recent United States Agency for International Developments Presidents Malaria Initiative (USAID PMI) report detailing Zambia IRS program costing, where M&E represented 6.2% of total program costs [17]. Authors chose the MINT model for this analysis given its ease of accessibility, including for MOH personnel, and its focus on IRS as a specific intervention.

### Cost for [IRS + Reveal]

The estimated ‘Total Cost of Ownership (TCO)’ for Reveal was estimated by applying a costing model and methodology developed by Digital Square for estimating all costs of developing and deploying digital platforms. This Excel-based tool was chosen for this analysis as it focuses on identifying common hidden costs, cost drivers and variances [19]. Authors applied the TCO model to summarize Reveal costs for the configuration and deployment of Reveal for one district (25 HFCs), and then scaled to all 125 HFCs across Luapula Province, Zambia over the course of 5 years. Development costs included costs for project management, needs assessment and requirements specifications as well as software development. These costs were constrained to year 1 and were captured from project budgets and reports. Deployment costs were considered annual costs spent for Reveal rollout during the yearly IRS campaign. These costs were captured also through project budgets and included equipment (cell phones), infrastructure (server), consulting services for implementation, software development time for any integrations or interoperability as well as estimates of costs for annual adaptation of the Reveal software, its testing and training. Development, deployment and operations costs are listed fully in Table 1.

**Table 1:** This table summarizes the TCO for Reveal across 5 years and 3 different dimensions including development and deployment (both considered 1-time costs) and operations (a recurring cost). The total 5-year cost of ownership was highest for year 1, considering start-up costs, and reduced for subsequent years

	Total costs per year (USD)					
Cost categories	Year 1	Year 2	Year 3	Year 4	Year 5	Total
**Development (one-time costs)**						
Project management	10 000					10 000
Needs assessment and requirements specifications	21 250					21 250
Software development	5400					5400
Subtotal	36 650	—	—	—	—	36 650
**Deployment (one-time costs)**						
Equipment	6550	6550	6550	5950	5950	31 550
Infrastructure	9600	9600	9600	9600	9600	48 000
Implementation services	3000	3000	3000	3000	3000	15 000
Geo-database building	4000	1000	1000	1000	1000	8000
Integration and interoperability	36 000	6000	6000	6000	6000	60 000
Software development and adaptation	4500	4500	4500	4500	2250	20 250
Testing	3500	3500	3500	3500	2500	16 500
New deployment training	15 500	8000	8000	8000	8000	47 500
Subtotal	82 650	42 150	42 150	41 550	38 300	246 800
**Operations (recurring costs)**						
Equipment replacement	2018	4035	6053	7920	9788	29 813
Infrastructure replacement	1920	3840	5760	7680	9600	28 800
Software licensing and subscriptions	—	—	—	—	—	—
Data and voice services	225	675	1125	1800	2700	6525
Microplanning processes	1000	1000	1000	1000	1000	5000
Refresher training	2000	2800	2800	2800	2800	13 200
Helpdesk support	5850	4250	4850	4250	4250	23 450
Maintenance	3750	3750	5000	6250	7500	26 250
Testing	2000	2000	2000	2000	2000	10 000
Transfer of ownership						—
Project management	12 000	8000	4000	4000	4000	32 000
Transportation and communication	2500	2500	3500	3500	3500	15 500
Governance	—	—	—	—	—	—
M&E	4000	4000	4000	4000	4000	20 000
Procurement	—	—	—	—	—	—
Total recurring (operations) costs	37 263	36 850	40 088	45 200	51 138	210 538
Total start-up (capital) costs	119 300	42 150	42 150	41 550	38 300	283 450
Total 5-year cost of ownership	156 563	79 000	82 238	86 750	89 438	493 988

Authors found the TCO model to include nearly all cost inputs necessary to fully describe the costs of Reveal deployment, including M&E costs. Each cost category was clearly described within the TCO model, and prompted for user input, and in some cases suggested input. Each input was fully described with user notes and guidance. One adaptation was made to the TCO model, and that was addition of line-item costs for building a geodatabase (basemaps) down to household level and supporting microplanning processes at provincial and district level. Current costs to configure and deploy Reveal were reported (costs for development of the open-source tool were excluded as these were considered sunk costs). The TCO costs for Reveal were then added to the IRS costs for a [IRS + Reveal] cost estimate. To ensure M&E costs were not factored in twice in the [IRS + Reveal] costs, the IRS cost was reduced by a factor of 6.2%, given that the TCO model for Reveal included associated M&E costs.

### Cases averted [IRS + Reveal]

Keating et al. (2021) conducted a retrospective cross-sectional study to provide evidence of epidemiological effectiveness of having introduced Actellic 300CS and Reveal (termed ‘mSpray’ at the time) into IRS programs across Zambia. Based on the incidence rate ratio (IRR) in that analysis was reported as (IRR = 0.83, 95% CI 0.72–0.95). This study then applied that IRR to calculate the improvement of adding Reveal to IRS delivery to report the additional Ca due to [IRS + Reveal] use in Luapula Province each year (as use of the Reveal tool scaled). The following equation was used to estimate Ca by [IRS + Reveal]: Ca [IRS + Reveal] = Ca [IRS only]/[IRR].

### Comparing the cost of [IRS only] to the cost of [IRS + Reveal]

Cost of [IRS only] per Ca was then compared to the cost of [IRS + Reveal] per Ca for Luapula Province for each year and across all 5 years.

## RESULTS

Results are reported first for the [IRS only] strategy followed by [IRS + Reveal].

### Estimate of cost and Ca by [IRS only]

Applying the census population of Luapula Province (1099151), the MINT tool was used to derive total Ca per year through IRS (*n* = 368 216) and the associated cost. The MINT tool reported the cost for [IRS only] in year 1 as $1 337 359 increasing to $6.7 million by year 5 (Table 2). For the [IRS only] strategy, cost per Ca was $18.16, which included 6.2% for M&E costs. These figures did not include any Reveal related costs.

### Estimate of cost and Ca by [IRS + Reveal]

**Figure 2:**
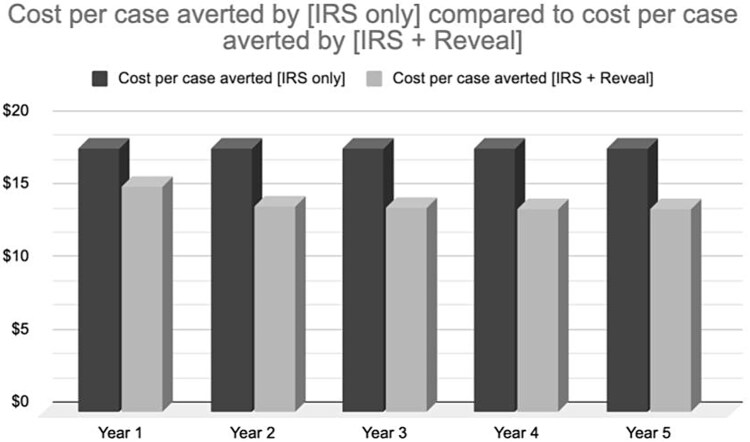
The cost per Ca is compared here between the [IRS only] and the [IRS + Reveal] strategy. The study found the [IRS + reveal] strategy represented a 15% reduction in cost per Ca in year 1, and 22%–23% lower average cost per Ca in years 2 to 5

Adding Reveal to the IRS campaign not only increased the total cost of the campaign each year but also increased the total number of Ca per year. Operation costs to deploy Reveal per year were estimated at $42 108 average per year; this cost ranged from $36 850 to $51 138 across the 5-year period, despite the significant scale across the entire province during that time (Table 1). Total 5-year cost of ownership was highest in year 1 ($156 563) decreasing to $79 000–$89 438 in subsequent years. For the [IRS + Reveal] strategy, cost per Ca was an average of $14.33 (range $13.93–$15.51). This represented a 15% reduction in cost per Ca (compared to [IRS only]) in year 1, increasing to a 23% reduction by years 3, 4 and 5 (Fig. 2). Further details on the Reveal cost results are provided below.

**Table 2:** This table summarizes the cost for [IRS only] compared to [IRS + Reveal] and the Ca for each strategy factored into the ‘cost per Ca’. Each year the [IRS + Reveal] strategy represented cost savings within a range of 15% to 23%

	Year 1	Year 2	Year 3	Year 4	Year 5
Scale HFC	25	25	25	25	25
Cumulative scale HFC	25	50	75	100	125
Estimated population	219 830	219 830	219 830	219 830	219 830
Cumulative population covered	219 830	439 660	659 491	879 321	1 099 151
Ca by [IRS only]	73 643	147 286	220 929	294 572	368 216
Cost for IRS	$1 337 359	$2 674 718	$4 012 077	$5 349 436	$6 686 795
Cost per Ca [IRS only]^*^	$18.16	$18.16	$18.16	$18.16	$18.16
Cost of Reveal	$156 563	$79 000	$82 238	$86 750	$89 438
Ca by [IRS + Reveal]	87 670	175 341	263 011	350 682	438 352
Cost for [IRS + Reveal]^*^^*^	$1 360 186	$2 486 246	$3 693 107	$4 901 242	$6 107 553
Cost per Ca [IRS + Reveal]	$15.51	$14.18	$14.04	$13.98	$13.93
[IRS + Reveal] cost savings	15%	22%	23%	23%	23%

^*^MINT model estimate, https://mint.dide.ic.ac.uk/; added 6.2% for M&E.

^**^Removed M&E cost from [IRS only] strategy to ensure no cost duplication for M&E.

All costs in USD.

### Reveal costing specifics

Development costs for the actual software application were considered sunk costs and not included in this analysis given that the platform was developed over the past 8 years through multiple funding sources and is now available for open-source use. However, costs for the initial stakeholder engagement and needs assessment and requirement specification work as well as minimal software development for setup (form updates and configuration) were considered ‘development costs’ and were included in year 1 at $36 650. These costs included project planning and scoping (travel costs and workshop costs) as well as a short needs assessment to define requirement specifications and gather map files. Software development staff (18 days) were also included to support data form modifications and limited changes to the web-based dashboard and application as well as the mobile client.

### Deployment costs

It included the infrastructure necessary to set up and conduct the implementation, support system interoperability, new deployment training and response to change requests. For equipment, 13 laptops were included (1 per each district added, plus 1 laptop for the provincial office). The model inputs estimated two health campaign supervisors per HFC, thus requiring a total of 50 mobile phones annually or 250 across 5 years. In this model, cloud-hosting was assumed although the application can be hosted locally. Thus, no servers were included, but rather monthly hosting costs of $800. Implementation services included costs for configuration of Reveal user accounts, settings and data forms resulting in an estimate of 20 person days each year at a local rate. Integration and interoperability cost estimates were made and front loaded into year 1, with maintenance costs included for years 2–5. One hundred and twenty (120) person-days (2 months of time for a team of 3 software engineers) was estimated to support integration. Integrations may be with existing health information systems already in use within a country. Software development and adaptation was also included to support new functionality requested during the implementation—here, a blended daily rate was utilized factoring in shifts toward local labor to support software (20 days for years 1–4 and 10 days in year 5). Testing costs were also included, which may include end-user experience tests, end-user acceptance tests, functionality tests in real-life settings and load (or stress/volume) tests. Ten days per year were included for testing at a local rate. Resources were included to conduct user acceptance testing workshops and/or on-site user testing (including travel, facility rental and per diems). Finally, resources for new deployment training and development/adaptation of training materials were included to support training of trainers in year 1 whereby master trainers would receive training to then subsequently train users across various levels of the province, district and health facility as the system scaled. In total, costs for deployment in Luapula Province were highest in year 1 at $82 650, and reducing by 49% in year 2 ($42 150), and stabilizing in subsequent years. These costs were reduced despite the scale of the system across the 5-year timeframe.

### Geodatabase building

Geodatabase building costs were added under the deployment cost category given the geo-functionality of Reveal requires building (or gathering) of initial basemap data assets. For this estimation, the use of freely-available geo resources, many of these derived from machine learning algorithms on satellite imagery was envisioned (Google Open Building dataset https://sites.research.google/open-buildings/, World Settlement Footprint data, Microsoft building footprints). A level of effort (3 days) for a geospatial information systems (GIS) technician to support geodatabase building in preparation for microplanning processes (using Reveal to support provincial and district processes) was also included.

## DISCUSSION

### Geospatial tooling is cost-effective

This study conducted a cost and cost-effectiveness analysis of the application of the digital geospatial tool (Reveal) added to a standard malaria control campaign (IRS) resulting in an estimation of the cost per Ca. Cost per Ca by the standard malaria control campaign [IRS only] was compared to costs per Ca through [IRS + Reveal]. Results indicated that addition of the geospatial tool to the standard IRS campaign resulted in cost savings. In year 1, cost per Ca of [IRS + Reveal] was $15.51 compared to IRS alone ($18.16), representing a 15% reduction in cost per Ca. Years 2–5 showed the [IRS + Reveal] strategy reporting a 22%–23% lower average cost per Ca compared to the standard malaria control campaign. The analysis suggests that the cost of adding geospatial digital tooling to IRS is cost-effective compared to the IRS intervention alone. Further, costs to deploy the geospatial tool reduced over time and as the tool scaled.

### Mechanisms for geospatial tooling impact

The high ROI reported for the [IRS + Reveal] strategy likely is attributed to two primary mechanisms: reduced redundancy and accelerated operations. IRS campaigns delivered through traditional M&E approaches—generally a combination of paper maps/forms and roaming supervision—often find field teams visiting structures multiple times during single campaigns, or missing structures altogether. Whether or not a structure has been sprayed is indicated by a paper form or chalk line affixed to a house (termed ‘structural attribution’), thus operators will only know if a house has been visited by visiting it. This approach can be problematic as the structural indication may disappear: paper blows away or chalk may become illegible. Taken together, these phenomena can increase the cost and reduce the impact of IRS delivery.

Use of geospatial technology for mapping and real-time visualization can provide field teams visual understanding of which structures are eligible for spraying, allowing field teams to navigate to sprayable structures more readily, and likely leading to higher ROI compared to standard approaches. Further, mapping structures prior to planning and IRS delivery more accurately determines the denominator making it clear where teams must deliver the intervention and ensuring that houses and populations are not missed. On average, field teams using the manual methods described above have been shown to only find approximately 55% of sprayable structures needing to be sprayed, and although reported coverage may be greater than 90%, actual coverage is closer to 49% [20]. Teams often miss clusters of structures in areas distant from higher population-dense places [21] and traditional IRS reporting only requires operators to report the number of sprayed structures as a percentage of the number of ‘found’ structures. Those operators only finding 50% of the structures needing to be sprayed in a particular geography inaccurately report 100% coverage if they were able to spray every structure they found. By using satellite-derived images of the target communities, program managers are not dependent on human factors to ‘find’ all the eligible structures. Eligibility is established from the imagery, and coverage of the spray campaign monitored through the platform. These considerations, although not directly tied to the ROI data, likely improve the efficiency of planning and delivery of IRS campaigns with Reveal.

### Global spending considerations

Extrapolating potential savings of geo-tooling to global spending on IRS further describes how application of Reveal could avert more cases per dollar spent. For example, the FY2024 US PMI [22] budget for Zambia allocates 2 million USD for IRS implementation and $800 000 for insecticides. Based on the ROI analysis reported here, use of the [IRS + Reveal] strategy would avert 14 027 more cases (or 15% more) in 1 year compared to IRS alone, based on the same spend. Of course numerous limitations must be factored in when extrapolating further, but as a loose benchmark, the global spend on health campaigns annually is $2.07 trillion USD [23]. If similar gains are achieved through use of geospatial technology to plan and deploy health campaigns at large, more than 19 million additional disease cases (or 15% more) could be averted based on the same spending levels.

### Approaches to scale

Several avenues exist to scale the tooling and future research should inform best practice approaches which likely include integration of the geospatial functionality to existing digital tools, particularly if these are already accepted and deployed widely in a country (e.g. integration of the microplanning module of Reveal to existing District Health Information System deployments (https://dhis2.org/)). Other avenues to scale could include integrated campaigns whereby multiple campaign types (e.g mass drug administration (MDA) for neglected tropical diseases, vaccines, Vitamin A) are all deployed using the same geospatial tooling, and data captured from households and their residents are used for subsequent campaign deployments. This type of scale should be explored and costed further, as well as re-use of the geospatial data from each campaign to further inform health and other information systems.

### Study limitations

The study did have several limitations. Cost inputs to the TCO model represented current estimated costs for deployment of the digital geospatial tool versus actual costs for implementation during the IRS campaign from which the incremental additional Ca data were extracted. Current costs were used (which represented an estimated 63% reduction compared to historic costs) as the platform has gained a significant degree of stability, maturity and platform enhancements over the past 3–5 years, which has resulted in reduced cost for deployment. These current costs were estimated based on similar sized, but recently conducted campaigns. Authors chose to use current costs considering these would be more relevant to countries or programs contemplating deploying the geospatial tool in the near future.

A second limitation was the use of modeled (MINT) estimates of Ca, and cost per Ca for the standard IRS intervention. The MINT model assumptions are fully detailed elsewhere (https://mint.dide.ic.ac.uk/), but in short, although the model was applied in Zambia, the original model was parameterized based on Tanzania data including the relative abundance of malaria vector species, the age distribution of the population and the relative effect of malaria interventions including the susceptibility of the mosquito vectors to insecticide products. A correlative analyses would be beneficial to explore similarities and differences between the Zambia and Tanzania model input variables.

The MINT model reported cost per Ca as $18.16; however, other estimations range as high as $105.15, meaning the cost per Ca related to the geospatial digital tool may be even more efficient. Also, for the Keating et al. (2021) evaluation, Reveal was deployed to areas with greater incidence of malaria relative to other areas of Zambia, meaning one may expect a higher number of Ca in those districts with or without the Reveal intervention. Another important note is that the costs within this analysis were extracted from one type of campaign and workflow (IRS) from specific a geography and with specific epidemiological trends. Caution must always be taken when applying these estimated costs and impacts to different types of campaign workflows and locations. For example, more time is required to configure the platform for a new campaign type increasing the cost for Reveal application particularly in year 1; or more mobile devices may be required for campaign deployment in some cases (see below); or the epidemiology of malaria or other diseases may respond differently to intervention coverage dynamics. In these cases, the TCO could be updated based on campaign requirements to refine costing estimates. Also, the option to deploy integrated campaigns raises the opportunity for cost savings by deploying the geospatial tooling across multiple campaigns simultaneously.

Several considerations are relevant to mention, including cost drivers related to digital tooling. One very significant cost driver is the requirement for phones or tablets for field team members. The number of devices can vary widely based upon the type of deployment model. For example, the IRS deployment model (which is the model used for the TCO in this analysis), required only two supervisors per HFC to be equipped with a mobile phone or tablet. However, other types of campaign deployments may require far more devices—such as mass drug administration which may rely on decentralized distribution teams operating in individual villages and required to complete the campaign in a short period of time to ensure entire populations are covered. In this model, individual village teams may require multiple devices, increasing the total device requirement up to 20–30 devices per health facility. In this case, increasing the number of devices to 20–30 per HFC would increase the TCO by approximately 60% per year.

For this analysis, the cost to purchase devices was included. However, recent deployments of Reveal have also relied on a ‘bring your own device’ (BYOD) model whereby field teams download the Reveal application to their personal devices. This approach may be useful to consider for large-scale deployments to control total costs. Further, many countries are moving toward a formalized community health worker (CHW) cadre that is equipped with phones for collection and submission of electronic health data into a centralized community health information system platform. In this case, phones would not be procured, except to fill any existing gaps, and Reveal would be downloaded to existing CHW phones, assuming the government’s minimum standard phone specification can support Reveal.

Often campaigns are deployed in extremely rural settings requiring alternative power for charging devices. For this analysis, infrastructure such as solar chargers and gensets was not included in the cost estimate. Another consideration and cost-driver is the prerequisite for geospatial basemaps to be built for import to the Reveal tool. For this analysis, moderate resources were included for basemap development. However, the level of effort required to develop basemaps may vary widely based upon availability of geospatial assets. In many areas, household footprint maps and models are readily available and increasingly sophisticated and free of charge. However, the quality and accuracy of these maps can vary significantly to the point that additional resources must be deployed for desk-top digitization or capture of other artificial intelligence–produced datasets. Also for consideration, Zambia uses sub-district targeting for IRS meaning that IRS is typically targeted to higher incidence areas, and/or based upon where ITN campaigns have been deployed. Use of a geospatial tool can inform the targeting strategy possibly contributing to a higher ROI, particularly in areas deploying targeted interventions. Use of geospatial tooling may not provide as strong an incremental cost effectiveness in areas, or for campaigns where a targeting approach is not applied. Future research is warranted.

## CONCLUSION

As country governments determine how best to allocate funds to achieve the SDGs, one key consideration is the best strategy and mix of interventions to achieve greatest impact and ROI. This analysis shows that the use of geospatial digital tooling to plan and deliver a health campaign bears a degree of cost effectiveness compared to the standard approach. Results indicate that geospatial tooling should be added to health campaign planning and delivery to improve the ROI of donor and government funds.

## Data Availability

Data available upon request.

## References

[1] Pryce J, Medley N, Choi L. Indoor residual spraying for preventing malaria in communities using insecticide-treated nets. *Cochrane Database Syst Rev* 2022;1:CD012688. 10.1002/14651858.CD012688.pub3 35038163 PMC8763033

[2] World Health Organization . Geneva. Operational Manual on Indoor Residual Spraying: Control of Vectors of Malaria, Aedes-Borne Diseases, Chagas Disease, Leishmaniases and Lymphatic Filariasis, 2023, https://iris.who.int/bitstream/handle/10665/375978/9789240083998-eng.pdf?sequence=1

[3] The Global Fund . *Malaria Report* 2024. https://www.theglobalfund.org/media/13753/publication_malaria_overview_en.pdf

[4] Larsen DA, Borrill L, Patel R et al. Reported community-level indoor residual spray coverage from two-stage cluster surveys in sub-Saharan Africa. *Malar J* 2017;16:249. 28610579 10.1186/s12936-017-1893-x PMC5470197

[5] Martin A, Bwalya F, Pollard D, et al. Use of a geo-enabled digital global good for microplanning and delivery of indoor residual spray (IRS) in Zambia: a case study, 2016–2020. Malar J. 2024; Under Review. 10.1371/journal.pgph.0004683 PMC12633927 41264575

[6] Keating J, Yukich JO, Miller JM et al. Retrospective evaluation of the effectiveness of indoor residual spray with pirimiphos-methyl (Actellic) on malaria transmission in Zambia. *Malar J* 2021;20:173. 33794892 10.1186/s12936-021-03710-5 PMC8017828

[7] Woods L, Eden R, Canfell O et al. Show me the money: how do we justify spending health care dollars on digital health? Med J Aust 218 2022;218:53–57. 10.5694/mja2.51799 PMC10107451 36502453

[8] Kolasa K, Kozinski G. How to value digital health interventions? A systematic literature review. *Int J Environ Res Public Health* 2020;17:2119. 10.3390/ijerph17062119 PMC7143608 32209988

[9] Trapp N, Schneider U, McCallum I et al. A meta-analysis on the return on Investment of geospatial data and systems: a multi-country perspective. *Trans GIS* 2014;09/01:169–187. 10.1111/tgis.12091

[10] Clements ACA, Reid HL, Kelly GC et al. Further shrinking the malaria map: how can geospatial science help to achieve malaria elimination? *Lancet Infect Dis* 2013;13:709–18. 23886334 10.1016/S1473-3099(13)70140-3

[11] Pigott DM, Howes RE, Wiebe A et al. Prioritising infectious disease mapping. *PLoS Negl Trop Dis* 2015;9:e0003756. 10.1371/journal.pntd.0003756 26061527 PMC4464526

[12] Ippolito MM, Gebhardt ME, Ferriss E et al. Scientific findings of the southern and Central Africa international center of excellence for malaria research: ten years of malaria control impact assessments in hypo-, meso-, and holoendemic transmission zones in Zambia and Zimbabwe. *Am J Trop Med Hyg* 2022;107:55–67. 36228903 10.4269/ajtmh.21-1287 PMC9662223

[13] Ferriss E, Chaponda M, Muleba M, et al. The impact of household and community indoor residual spray coverage with Fludora fusion in a high malaria transmission setting in northern Zambia. Am J Trop Med Hyg Aug 2023;109:248–57. 37364860 10.4269/ajtmh.22-0440 PMC10397455

[14] United States agency for international development. Luapula Province Fact Sheet 2021. 1–3. https://www.usaid.gov/sites/default/files/2022-05/FINAL_Luapula_Province_Fact_Sheet_Dec._2021.int_.pdf

[15] Das S, Muleba M, Stevenson JC et al. Habitat partitioning of malaria vectors in Nchelenge District, Zambia. *Am J Trop Med Hyg* 2016;94:1234–44. 27001755 10.4269/ajtmh.15-0735 PMC4889739

[16] Muleba M, Mbata KJ, Stevenson JC et al. Spatial-temporal vector abundance and malaria transmission dynamics in Nchelenge and Lake Mweru islands, a region with a high burden of malaria in northern Zambia. *Malar J* 2023;22:327. 37899457 10.1186/s12936-023-04746-5 PMC10613358

[17] Aghajanyan A, Riley M, Tesso E et al. *Comparative Cost Analysis*. PMI IRS Country Programs, 2023;2022:1–84.

[18] Measure Evaluation . How Much Do Evaluations and Other Surveys Cost? University North Caroline Chapel Hill. 2019. https://www.measureevaluation.org/resources/publications/fs-15-156/at_download/document

[19] PATH . Digital Square. *Total Cost of Ownership Tool* 2022. 1–3. https://digitalsquare.org/tco-tool

[20] Bridges DJ, Pollard D, Winters AM et al. Accuracy and impact of spatial aids based upon satellite enumeration to improve indoor residual spraying spatial coverage. *Malar J* 2018;17:93. 29471832 10.1186/s12936-018-2236-2 PMC5824454

[21] Mackowski M . Factors Limiting Effective Coverage of Indoor Residual Spraying Campaigns in Luapula Province. Zambia: Syracuse University, 2020, https://surface.syr.edu/thesis/400/?utm_source=surface.syr.edu%2Fthesis%2F400&utm_medium=PDF&utm_campaign=PDFCoverPages

[22] U.S. President’s Malaria Initiative Zambia Malaria Operational Plan FY 2024. 2023. www.pmi.gov

[23] Camber Collective . Campaign Effectiveness Landscape and Case for Action*.* Seattle. 2020. https://taskforce.org/wp-content/uploads/2020/04/Campaign-Effectiveness-Landscape-and-Case-for-Action-February-2020-Public.pdf

